# The Information Technology Aided Relapse Prevention Programme in Schizophrenia: an extension of a mirror-design follow-up

**DOI:** 10.1111/j.1742-1241.2008.01903.x

**Published:** 2008-12

**Authors:** F Španiel, P Vohlídka, J Kožený, T Novák, J Hrdlička, L Motlová, J Čermák, C Höschl

**Affiliations:** 1 Prague Psychiatric CenterPrague, Czech Republic; 2 3rd Faculty of Medicine, Charles University PraguePrague, Czech Republic; 3 Center of Neuropsychiatric StudiesPrague, Czech Republic; 4 Eli Lilly and Company LimitedBasingstoke, Hampshire, UK; 5 Department of Cybernetics, Faculty of Electrical Engineering, Czech Technical UniversityPrague, Czech Republic

## Abstract

**Aims:**

Decreasing a number of hospital admissions is important for improving outcomes for people with schizophrenia. The Information Technology Aided Relapse Prevention Programme in Schizophrenia (ITAREPS) programme enables early pharmacological intervention in psychosis by identification of prodromal symptoms of relapse using home telemonitoring via a phone-to-PC SMS platform.

**Methods:**

This study was a 1-year extension of a previously published mirror-design follow-up evaluation of programme clinical effectiveness. In total, 73 patients with psychotic illness (45 patients from original sample and 28 newly added subjects) collaborating with 56 family members participated in the clinical evaluation.

**Results:**

There was a statistically significant 77% decrease in the number of hospitalisations during the mean 396.8 ± 249.4 days of participation in ITAREPS, compared with the same time period before participation in ITAREPS (Wilcoxon-signed ranks test, p < 0.00001), as well as significantly reduced number of hospitalisation days when in the ITAREPS (2365 hospitalisation days before and 991 days after ITAREPS enrolment respectively, Wilcoxon-signed ranks test, p < 0.003).

**Conclusion:**

The ITAREPS programme represents an effective tool in the long-term treatment of patients with psychotic disorders.

**What's known**

Studies published so far suggest that crisis interventions such as increasing the dose of antipsychotic medication, based on early detection within a relapse prevention programme, reduce readmission rates in schizophrenia. Previous findings from ITAREPS mirror-image design follow-up showed a statistically significant 60% decrease in the number of hospitalisations during the mean 283.3 ± 111.9 days of participation in ITAREPS compared with the same time period before entering the programme (sign test, p < 0.004).

**What's new**

The ITAREPS enables early pharmacological intervention in psychosis by identification of prodromes of relapse using home telemonitoring via a phone-to-PC SMS platform. ITAREPS demonstrated sustained effectiveness for 1 year after the conclusion of the previous mirror-image design follow-up, decreasing the total number of hospitalisations by 77% and the number of hospitalisation days by 58% respectively, during the time spent in ITAREPS compared with a period of equal length prior to ITAREPS enrolment.

## Introduction

The need for providing more efficient healthcare services, coupled with major advancements in information and high-tech communication technology, has led to novel approaches to medical treatment during the last decade. E-health technologies combine the use of electronic communication and information technology in the healthcare for clinical, education and administrative purposes.

The Information Technology Aided Relapse Prevention Programme in Schizophrenia (ITAREPS), developed by the Prague Psychiatric Centre in the Czech Republic, presents a mobile phone-based e-health solution for remote patient monitoring and disease management in schizophrenia and psychotic disorders in general.

The main incentive behind ITAREPS development was emerging evidence that acute psychotic state may result in certain pathophysiological processes in the central nervous system (CNS) responsible for deterioration of the overall clinical condition (1). Increased number of psychotic relapses contribute to a social decline and can interfere with a treatment response (2–4). Moreover, available data suggest correlation of increased relapse/hospitalisation rate with structural abnormalities of the CNS (5). There is also convincing evidence of an association between duration of the untreated psychosis and adverse clinical outcome, which supports the notion of toxic effect of psychotic state (6,7).

These findings emphasise the need for a decrease in the number of relapses as one of the main goals of long-term treatment of psychotic illness.

The Information Technology Aided Relapse Prevention Programme in Schizophrenia-mediated telemonitoring identifies early warning symptoms of an illness. It enables early pharmacological intervention and helps to prevent relapses and recurrent hospitalisations. Previous findings from a mirror-image design (i.e. one group, uncontrolled, non-blind, before-after design) follow-up evaluation of clinical effectiveness of the ITAREPS programme in 45 patients with psychotic illness (8) showed a statistically significant 60% decrease in the number of hospitalisations during the mean 283.3 ± 111.9 days of participation in ITAREPS compared with the same time period before entering the programme (sign test, p < 0.004).

The main aim of this clinical evaluation was to confirm sustained effectiveness of the programme, one additional year after the conclusion of the previous mirror-image design follow-up.

## Methods

Each participant, a patient and her/his family member enrolled in the ITAREPS programme, completes a 10-item Early Warning Signs Questionnaire (EWSQ, patient and the family member version respectively). Reporting on psychometric properties and structure of EWSQ has been published elsewhere (8,9). Reminder for the completion of the EWSQ is sent weekly by an automated system to their mobile phones as an SMS. Individual EWSQ scores are sent by participants back to ITAREPS as an SMS. No specific training is provided for ITAREPS participants except information detailed in the ITAREPS user guide distributed to the programme participants.

If a total EWSQ score exceeds a given score threshold, an automatically generated ALERT is declared and a treating psychiatrist is notified by an e-mail message. According to the Early Intervention Algorithm (EIA), a part of ITAREPS, the presence of early warning signs warrants an immediate increase in baseline maintenance dose of antipsychotic by 20% within the next 24 h. However, data about nature of early intervention were not captured.

An ITAREPS web-based interface (http://www.itareps.com) offers an authorised physician, a user-friendly process to enter patient data. It also provides summaries and continuous reporting on current clinical status of each patient participating in the ITAREPS.

A total of 73 outpatients with psychotic disorders were enrolled by their psychiatrists through 25 outpatient facilities in the Czech and Slovak Republics for routine clinical use of the programme. Forty-five patients completed previously published study (8). Additional 28 patients were added to this cohort.

There were no qualifying criteria for inclusion except the diagnosis of psychotic illness. Patients fulfilled International Classification of Diseases (ICD)-10 criteria for diagnosis of schizophrenia, schizoaffective disorder or acute polymorphic psychotic disorder with or without symptoms of schizophrenia (Table 1). Of the 73 patients, 56 had a participating family member. Participation of a family member in ITAREPS was highly recommended, albeit optional.

**Table 1:** The demographic and clinical characteristics of the patients

	Mean (SD)
Age	
Male (*n* = 40)	29.9 (6.7) years
Female (*n* = 33)	29.5 (9.4) years
Baseline CGI	2.7 (1.2)
Duration of illness	7.9 (5.3) years
Number of hospitalisations beforeITAREPS entry	2.9 (3.3)
	*n*
Diagnosis	
Schizophrenia	47 (64.4%)
Schizoaffective disorder	15 (20.5%)
Acute polymorphic psychotic disorder with schizophrenia symptoms	10 (13.7%)
Acute polymorphic psychotic disorder without schizophrenia symptoms	1 (1.4%)
Antipsychotic medication	
No	5 (6.9%)
Yes	68 (93.1%)

ITAREPS, Information Technology Aided Relapse Prevention Programme in Schizophrenia; CGI, Clinical Global Impression.

The following baseline patient data were obtained and entered by psychiatrists: demographic data; diagnosis; illness history; Clinical Global Impression Severity Scale (CGI-S) and current medication. Compliance was evaluated by psychiatrists using a questionnaire with a 5-point scale [one = always and five = never taking antipsychotic medication (10)].

As this follow-up used only clinical information without specific patient identifiers and the procedures required no deviation from standard clinical practice, informed consent was not obtained from participants. The protocol of ITAREPS programme was approved by the Ethics Committee of the Prague Psychiatric Centre.

## Results

The number of hospitalisations was assessed individually for each patient during the period of her/his participation in ITAREPS programme compared with equal period of time prior to ITAREPS enrolment.

In total, there were 14 hospitalisations during a mean of 396.8 ± 249.4 days follow-up compared with 60 hospitalisations during the same length of time before entering the programme. The results showed a statistically significant reduction of 77% in the number of hospitalisations during the ITAREPS programme participation (Wilcoxon-signed ranks test, Monte Carlo, exact test, two-tailed, *Z* = 4.86, p < 0.00001).

There was a statistically significant reduction of 58% in the number of hospitalisation days during the programme participation compared with the mirror period before the ITAREPS entry (in total 2365 hospitalisation days before the ITAREPS compared with 991 days after the programme entry respectively, Wilcoxon-signed ranks test, Monte Carlo, exact test, two-tailed, *Z* = 2.86, p < 0.003).

There was 100% decrease in the number of hospitalisations (from 25 to zero) in patients within highly co-operative patient/family member pairs (*n*= 25, 34% of all patients) during the follow-up. The participants′ high co-operation was set up as at least 70% of the expected SMS with the EWSQ rating were delivered to the system.

This finding remains unchanged from the previous follow-up results where 100% decrease in the number of hospitalisations (from 13 to zero) was observed in patients from highly co-operative patient/family member pairs (*n* = 21, 47% of all patients).

No significant difference was found between patients with hospitalisation and patients without hospitalisation in the ITAREPS programme regarding duration of illness, number of hospitalisations before entering the ITAREPS, CGI-S rating at the baseline ITAREPS visit, medication compliance, medication formulation (depot vs. oral antipsychotic medication), length of participation in the ITAREPS programme, number of ALERT messages, involvement of a family member or separately assessed co-operation of a patient or a family member respectively. A controlled randomised study should be warranted to find out what effect these variables have on effectiveness of ITAREPS.

However, the group of patients without hospitalisation showed a significantly higher proportion of highly co-operative patient/family member pairs compared with subjects with hospitalisation during ITAREPS programme participation (Wilcoxon-signed ranks test, Monte Carlo, exact test, two-tailed, p = 0.009, test-specific α error level 0.015 – Tukey’s adjustment for 11 primary end-points).

## Discussion

The effectiveness of the ITAREPS programme, in conjunction with its low set-up and operating costs, makes ITAREPS an attractive option in the long-term treatment and management of patients with schizophrenia and psychotic disorders in general. Albeit user-friendly and easy-to-understand system with minimal eligibility requirements for patient implying its utilizability in common clinical settings, the programme applicability in severely ill, less compliant subjects warrants further investigation.

The results of the clinical follow-up should be interpreted with caution because of methodological limitations of the mirror-image design (the absence of a control group, lack of blinding and randomisation, inability to control for factors like patients’ decreasing risk of rehospitalisation over time). In addition, although a specific pharmacological intervention was recommended according to EIA, the exact nature of the intervention during the ALERT states could not be documented in this clinical evaluation.

Therefore, a prospective, double-blind, randomised trial with the ITAREPS programme is warranted to confirm these promising findings.
